# Mediating Effect of Attitude Between Knowledge and Willingness to Donate and Accept Donor Breast Milk in Odisha, India

**DOI:** 10.7759/cureus.109709

**Published:** 2026-05-26

**Authors:** Hepsi Bai Joseph, Sandhiya K, Reeya Majhi, Riya Mandal, Ruchi Patel, Ms Roshna, Sananda Chakraborty, Jaison Jacob

**Affiliations:** 1 Child Health Nursing, All India Institute of Medical Sciences, Bhubaneswar, Bhubaneswar, IND; 2 Nursing, All India Institute of Medical Sciences, Bhubaneswar, Bhubaneswar, IND

**Keywords:** acceptance, attitude, awareness, milk banks, milk donation, mothers

## Abstract

Introduction: Breast milk banking (BMB) serves as a vital intervention to support neonatal health, particularly for preterm and low-birth-weight infants. However, cultural, attitudinal, and knowledge-based barriers affect its acceptance. This study assessed mothers' knowledge, attitudes, and willingness regarding breast milk donation (BMD) and donor breast milk (DBM), exploring factors influencing these aspects and the mediating role of attitude.

Methods: A cross-sectional analytical study was conducted from November 2021 to July 2022, involving 400 mothers selected through convenience sampling. Data were collected using validated questionnaires assessing demographic details, knowledge, attitudes, and willingness toward BMB. Descriptive statistics, correlation, chi-squared tests, prevalence ratios, and mediation analysis using SmartPLS (SmartPLS GmbH, Bönningstedt, Germany) were performed.

Results: Most participants, 250 (62.5%), were unaware of BMB, though 259 (64.8%) showed a favorable attitude. Willingness to donate was reported by 313 (78.3%), while 279 (69.8%) were willing to accept DBM. Major barriers included family disapproval and fear of disease transmission. Knowledge positively correlated with willingness but negatively with attitude. Mediation analysis showed that while higher knowledge directly increased willingness, it also reduced favorable attitudes, which negatively impacted willingness. Socio-demographic factors such as education, income, residence, and family type significantly influenced knowledge, attitudes, and willingness.

Conclusion: Although mothers with higher knowledge were more willing to donate and accept breast milk, this was paradoxically linked to less favorable attitudes. Addressing misconceptions and promoting community-based education may improve both attitudes and participation in BMB programs.

## Introduction

Breastfeeding is universally recognized as the optimal source of infant nutrition, providing essential nutrients, immune protection, and developmental benefits [1]. Exclusive breastfeeding can prevent 13% of under-five deaths globally every year [2,3]. However, for various reasons, such as maternal health issues, insufficient milk supply, or the demands of premature births, not all mothers can breastfeed [4,5]. In these situations, donor breast milk (DBM) can serve as an essential alternative, offering similar health benefits and reducing the risk of infant mortality and diseases. While the best option is the mother's milk, DBM is the next most beneficial alternative when the mother cannot breastfeed.

To ensure equitable access to safe human milk, breast milk banks (BMBs) play a critical role by collecting, screening, processing, and distributing excess milk from lactating mothers [6]. While several countries have integrated milk banks into national nutrition strategies [7,8], in India, despite over 90 functional BMBs, the awareness and utilization of donor milk remain limited, especially in rural and underserved areas [6]. Odisha, a state with high rates of low birth weight and neonatal mortality, established its first milk bank only recently in 2022 [9]. Given the novelty of this intervention, mothers' decisions to donate or accept breast milk are likely influenced by personal beliefs, cultural norms, and a lack of information [10,11].

Mothers' willingness to donate breast milk or accept DBM may be influenced by their knowledge and attitudes toward BMB [12]. In culturally sensitive settings such as India, increased awareness alone may not necessarily translate into willingness unless accompanied by favorable attitudes toward the practice [13]. Therefore, attitude may serve as a key mediating factor between knowledge and behavioral intentions regarding breast milk donation (BMD) and acceptance. 

This study aimed to assess mothers' knowledge, attitudes, and willingness regarding BMB and to examine whether attitude mediates the association between knowledge and willingness toward BMD and acceptance of DBM in Odisha, India. By employing the Theory of Planned Behavior (TPB), which posits that intention to perform a behavior is influenced by three primary factors, namely, attitude, subjective norms, and perceived behavioral control, we explore how knowledge about BMB affects mothers' attitudes and how it shapes their willingness to donate and accept DBM [14].

## Materials and methods

Conceptual framework: TPB

**Figure 1:**
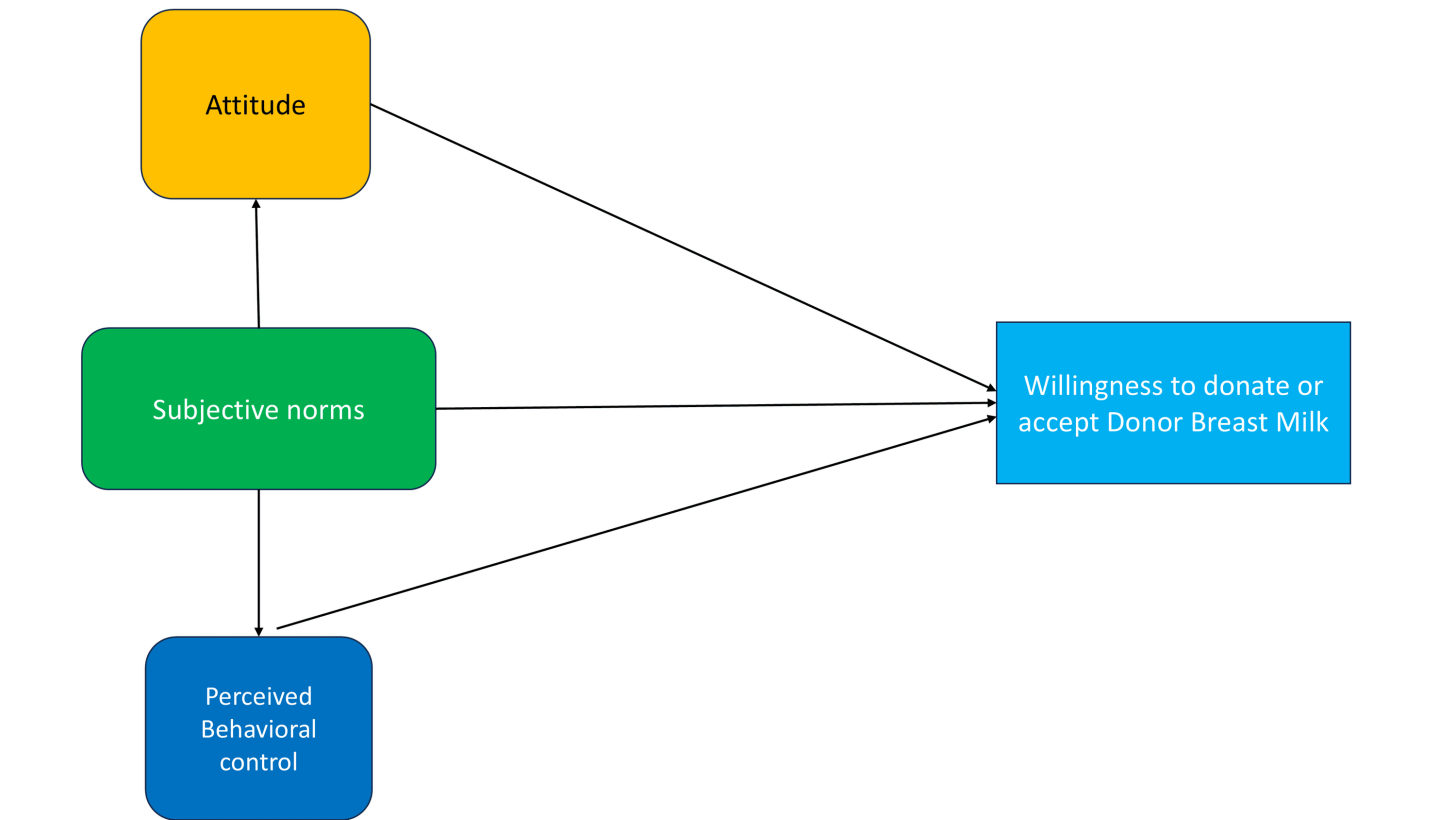
Conceptual framework illustrating the mediating role of attitude between knowledge and willingness to donate and accept donor breast milk based adapted from the Theory of Planned Behavior Image created by the authors using Microsoft PowerPoint (Microsoft Corporation, Redmond, Washington, United States)

TPB provides a valuable lens for understanding this decision-making process [14]. According to TPB, intention to perform a behavior is influenced by three main components: attitude (positive or negative evaluation of the behavior), subjective norms (perceived social expectations), and perceived behavioral control (belief in one's ability to perform the behavior) (Figure 1).

Subjective norms were indirectly reflected through socio-demographic and family-related characteristics, while knowledge and resource-related factors may influence perceived behavioral control regarding BMD and acceptance. In the present study, attitude was examined as the primary proximal determinant linking knowledge with willingness regarding DBM.

In this context, mothers' knowledge about milk banking may not directly lead to action unless accompanied by a favorable attitude toward donation. This highlights the importance of exploring whether attitude mediates the relationship between knowledge and willingness to donate breast milk.

This study aims to examine the mediating effect of attitude between knowledge and willingness regarding BMD and acceptance of DBM among mothers in Odisha, using the TPB as the guiding framework.

The following are the hypotheses developed and tested in the current study: H1: mothers with higher knowledge about BMB are more likely to express willingness for BMD and acceptance of DBM; H2: mothers with favorable attitudes toward BMB are more likely to express willingness for BMD and acceptance of DBM; H3: knowledge about BMB is positively associated with favorable attitudes toward BMB; H4: attitude mediates the relationship between knowledge and willingness for BMD and acceptance of DBM; and H5: socio-demographic variables (such as education, income, and family type) are significantly associated with knowledge, attitudes, and willingness for BMD and acceptance of DBM.

Participants

A cross-sectional analytical study was conducted with mothers (antenatal and postnatal) using a convenience sampling approach at All India Institute of Medical Sciences, Bhubaneswar, a tertiary care center in Bhubaneswar, Odisha, India, which caters to patients from across the state and neighboring regions. Ethical approval was obtained from the Institutional Ethics Committee of All India Institute of Medical Sciences, Bhubaneswar (approval number: T/IM-NF/Nursing/21/139), and the study lasted from November 2021 to July 2022.

Eligible antenatal and postnatal mothers present during the data collection period were approached consecutively by trained researchers and invited to participate. The study objectives were explained, and written informed consent was obtained prior to enrolment. Mothers who were critically ill, unable to communicate, or unwilling to participate were excluded. A small number of eligible mothers declined participation due to a lack of time or personal reasons.

Power analysis and sample size

A priori power analysis was conducted using G*Power Version 3.1 (Heinrich-Heine-Universität Düsseldorf, Düsseldorf, Germany) to determine the minimum required sample size for mediation analysis using multiple linear regression. With an effect size (f²) of 0.05 (indicating a small effect), a significance level (α) of 0.05, and a power of 0.95, the total number of predictors was set at nine, including demographic variables. Among these, four were tested predictors, representing key components in the hypothesized mediation model. Based on these inputs, the estimated minimum sample size required was 377 participants. Hence, a total of 400 participants were included in the study to ensure sufficient statistical power to detect meaningful relationships among the variables, including direct and indirect effects.

Instruments used (Appendix A)

Demographic Variables

The demographic questionnaire consisted of nine items assessing participants' age, residence, marital status, religion, educational status, occupation, monthly family income, type of family, and number of children.

Knowledge Questionnaire on BMB

The knowledge questionnaire was developed based on a literature review and expert consultation and consisted of 15 multiple-choice questions with one correct response for each item. Total scores ranged from 0 to 15, with scores of 0-5 indicating low awareness, 6-10 indicating moderate awareness, and 11-15 indicating high awareness regarding BMB and donation. The categorization was based on expert consensus during tool development.

The questionnaire was pretested among five antenatal and five postnatal mothers to assess clarity, comprehensibility, and cultural appropriateness. Feedback obtained during pretesting was incorporated into the final version. Content validity was evaluated by five experts from the departments of Neonatology, Obstetrics and Gynecology, and Nursing, yielding a content validity index (CVI) of 0.85. Reliability was assessed using the split-half method and found to be 0.85 [15]. 

Attitude Rating Scale on BMB

Attitude toward BMB was assessed using a 14-item, 5-point Likert rating scale developed through literature review and expert consultation. Items 1-7 were positively worded and scored as follows: strongly agree=5, agree=4, neutral=3, disagree=2, and strongly disagree=1. Items 8-14 were negatively worded and reverse-scored. Total attitude scores ranged from 14 to 70, with higher scores indicating more favorable attitudes toward BMB. Scores of 43-70 were categorized as favorable attitudes, while scores of 14-42 indicated unfavorable attitudes. The cutoff values were determined based on the possible score range and expert recommendations during tool validation. Reliability of the scale was established using the test-retest method and was found to be 0.88.

Willingness Questionnaire

The willingness questionnaire consisted of two items assessing mothers' willingness to donate breast milk and accept DBM, along with open-ended questions exploring reasons for unwillingness. The questionnaire was developed based on a literature review and expert input and underwent face and content validation as part of the overall tool validation process. All study instruments were translated into Odia and reviewed for semantic and cultural appropriateness before data collection.

Plan for data collection

Data were collected by trained researchers who administered the questionnaires to the participants. Before filling out the questionnaires, participants provided informed consent. The researchers ensured that the collected data were complete and accurate by carefully checking the questionnaires for completeness after they were filled out.

Statistical analysis

Statistical analysis was performed using IBM SPSS Statistics for Windows, Version 21.0 (IBM Corp., Armonk, New York, United States). Descriptive statistics were used to summarize socio-demographic characteristics and study outcomes. The Mann-Whitney U test examined associations between demographic variables and study outcomes, while Spearman's rank correlation coefficient (Spearman's rho) evaluated correlations between knowledge, attitudes, and willingness regarding BMD and acceptance of DBM. The chi-squared test and prevalence ratios (PR) with 95% confidence intervals (CI) were used to identify factors affecting willingness to donate or accept donor milk across educational, family type, income, knowledge, attitude, and religious categories.

Partial least squares structural equation modeling (PLS-SEM) was conducted using SmartPLS Version 4.1.1.2 (SmartPLS GmbH, Bönningstedt, Germany) to examine the mediating role of attitude in the relationship between knowledge and willingness regarding BMD and DBM. A reflective measurement model was specified, with knowledge and attitude treated as latent constructs, while the willingness outcomes were treated as observed variables. Bootstrapping with 5,000 resamples was performed to estimate the significance of direct and indirect effects. Measurement model evaluation included assessment of indicator loadings, internal consistency reliability (composite reliability), convergent validity (average variance extracted), and discriminant validity using the heterotrait-monotrait (HTMT) ratio and Fornell-Larcker criterion. Structural model assessment included collinearity diagnostics, path coefficients, coefficient of determination (R²), and significance of mediation effects. Detailed measurement model results are provided in Appendix B-Appendix G. Cases with incomplete responses were excluded prior to analysis, and only complete datasets were included in the final model.

## Results

 Socio-demographic characteristics of the mothers

The socio-demographic details of the 400 participating mothers revealed that the majority were aged 26-35 years, 204 (51%), with a mean age of 26.45±4.17 years, and 230 (57.5%) resided in rural areas. All participants were married 400 (100%). Most mothers were Hindu, 356 (89%), and had graduate-level education, 140 (35%), and 258 (64.5%) were unemployed or homemakers. The largest income group earned Rs. 5,000-20,000 per month, 207 (51.75%). Additionally, 203 (50.75%) lived in joint families, and 222 (55.5%) had one child. This socio-demographic profile is important for understanding their views on BMD and BMB.

Level of knowledge and attitude regarding BMB

**Figure 2:**
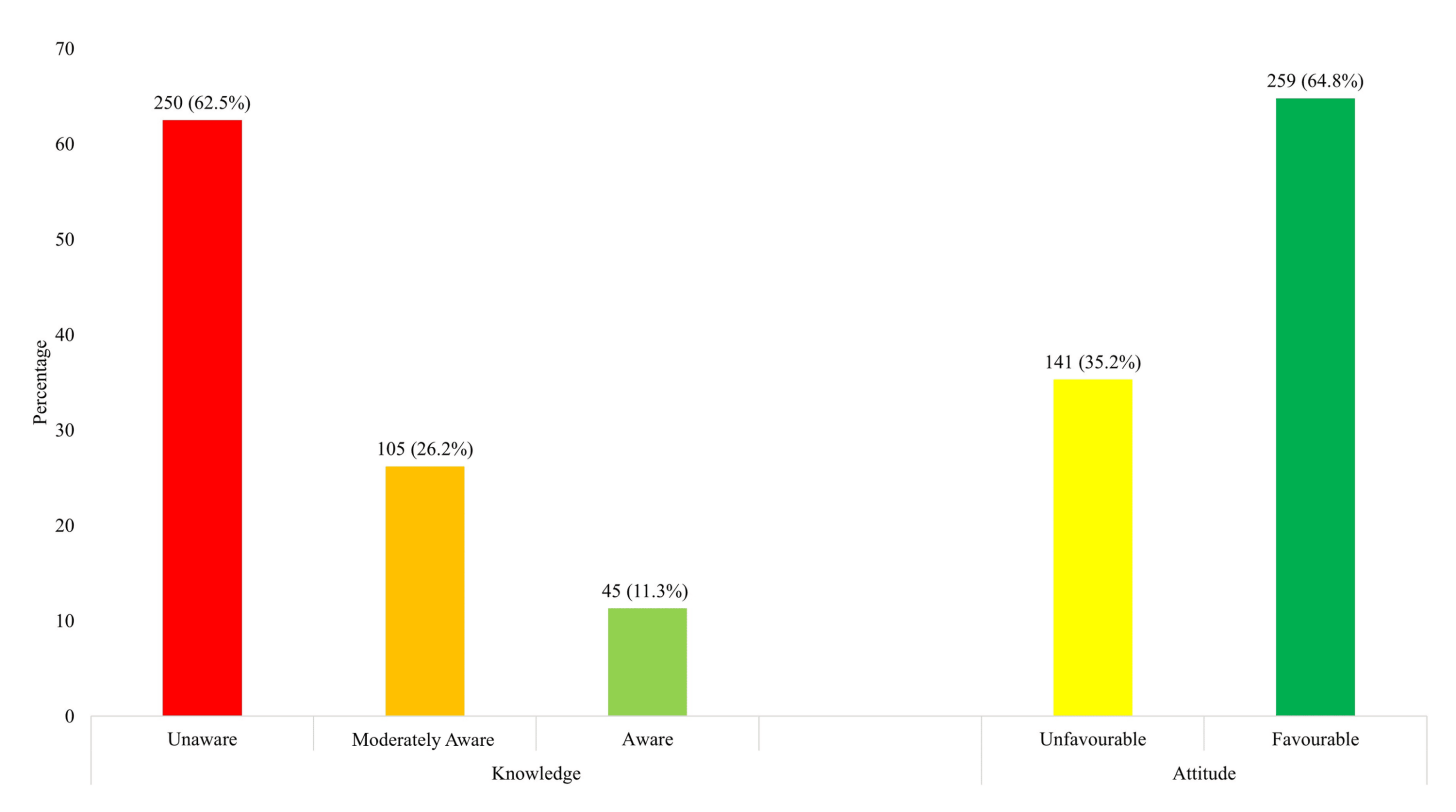
Level of knowledge and attitude regarding breast milk donation and banking (n=400) Image created by the authors using Microsoft PowerPoint (Microsoft Corporation, Redmond, Washington, United States)

Figure 2 shows that 250 (62.5%) mothers were unaware, 105 (26.25%) were moderately aware, and 45 (11.25%) were aware of BMD and BMBs. Additionally, 259 (64.8%) mothers had a favorable attitude, while 141 (35.2%) held an unfavorable attitude towards BMD and BMBs.

Willingness to donate and accept DBM and reasons for non-willingness

**Figure 3:**
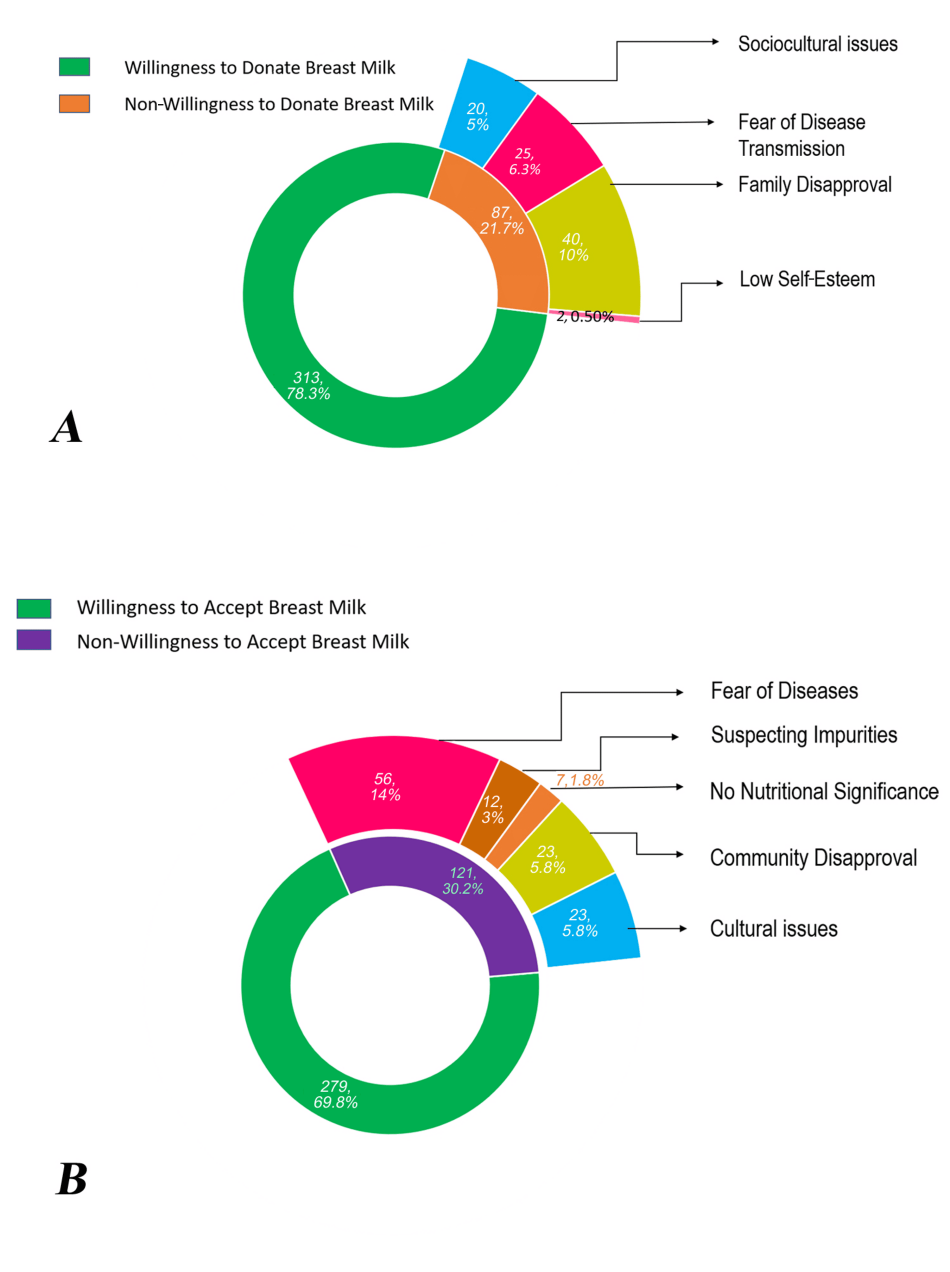
Willingness to (A) donate and (B) accept donor breast milk (n=400) Image created by the authors using Microsoft PowerPoint (Microsoft Corporation, Redmond, Washington, United States)

Figure 3 presents mothers' willingness for BMD and to accept DBM.

In Figure 3A, 313 (78.3%) mothers were willing to donate breast milk, while 87 (21.7%) were not. The main reasons for unwillingness included family disapproval, 40 (10%), fear of disease transmission, 25 (6.3%), sociocultural issues, 20 (5%), and low self-esteem, 2 (0.5%).

In Figure 3B, 279 (69.8%) mothers were willing to accept DBM, whereas 121 (30.2%) were not. The key reasons for unwillingness were fear of diseases, 56 (14%), community disapproval, 23 (5.8%), cultural issues, 23 (5.8%), suspected impurities, 12 (3%), and no nutritional significance, 7 (1.8%).

The reasons for unwillingness are calculated from the subgroup of mothers who were not willing (n=87 for donation; n=121 for acceptance), and frequency and percentage are based on the overall number of 400.

Correlation analysis of knowledge, attitude, and willingness

**Figure 4:**
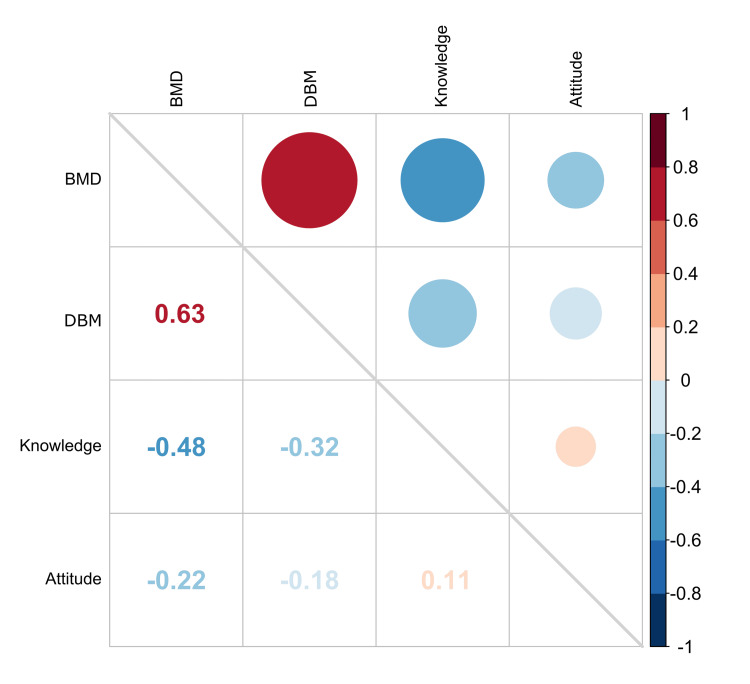
Correlation matrix of knowledge, attitude, and willingness to donate and accept DBM Spearman's rank correlation coefficient was used to assess correlations among the study variables. The color bar represents the strength and direction of the correlation (ρ values). Circle size indicates the magnitude of the correlation. BMD: breast milk donation; DBM: donor breast milk Image created by the authors using Microsoft PowerPoint (Microsoft Corporation, Redmond, Washington, United States)

Figure 4 demonstrates the correlations among knowledge, attitude, and willingness outcomes. Knowledge showed a weak but statistically significant positive correlation with attitude toward BMB (ρ=0.11; p<0.05). Knowledge was negatively correlated with willingness to BMD (ρ=-0.48; p<0.01) and willingness to accept DBM (ρ=-0.32; p<0.01). Attitude was negatively correlated with willingness for BMD (ρ=-0.22; p<0.05) but positively correlated with willingness to accept DBM. A strong positive correlation was observed between willingness to donate and willingness to accept DBM (ρ=0.63; p<0.01).

Determinants of willingness to donate and accept DBM

Table 1 reveals several significant factors associated with the likelihood of willingness to donate breast milk. Graduates were significantly more likely to donate than non-graduates (PR=1.14; 95% CI: 1.03-1.26), as were individuals with a monthly income above ₹20,000 (PR=1.15; 95% CI: 1.04-1.27). Rural residents also showed a significantly higher likelihood of willingness to BMD (PR=1.52; 95% CI: 1.28-1.80). Participants from joint families were significantly less likely to donate breast milk compared to those in nuclear families (PR=0.87; 95% CI: 0.78-0.96), and Hindu participants were less likely to donate breast milk than others (PR=0.82; 95% CI: 0.74-0.90). Notably, those who were not aware of BMD had a significantly higher likelihood of donating (PR=2.62; 95% CI: 2.00-3.43).Variables with no significant association with willingness to donate included occupation, attitude, and number of children.

**Table 1:** PR for willingness to donate and accept DBM by socio-demographic, knowledge, and attitudinal factors BMD: breast milk donation; DBM: donor breast milk; PR: prevalence ratio; CI: confidence interval *p<0.05 and **p<0.01: statistically significant

Variable	Willingness to BMD			Willingness to DBM		
	Yes/no	ꭓ^2^ value	PR (95% CI)	Yes/no	ꭓ^2^ value	PR (95% CI)
Mothers/pregnant women						
1 or more child	234/56	3.688	1.12* (0.98-1.27)	207/83	1.327	1.31* (0.82-2.10)
No child	79/31			72/38		
Education						
Graduates	144/27	6.23*	1.14 (1.03-1.26)	139/32	18.84**	1.33* (1.17-1.50)
Non-graduates	169/60			140/89		
Occupation						
Housewives	203/55	0.08	1.01* (0.91-1.13)	169/89	6.21*	0.84* (0.74-0.95)
Employed	110/32			110/32		
Family						
Joint	148/55	6.91**	0.87* (0.78-0.96)	131/72	5.31*	0.85* (0.75-0.97)
Nuclear	165/32			148/49		
Income (INR)						
>20000	144/26	7.24**	1.15* (1.04-1.27)	127/43	3.44	1.13* (0.99-1.28)
Below 20000	169/61			152/78		
Religion						
Hindu	273/84	6.17*	0.82* (0.74-0.90)	243/114	4.45*	0.81* (0.70-0.94)
Others	40/3			36/7		
Attitude						
Favorable	148/111	12.81**	0.76 (0.65-0.87)	200/59	4.25*	0.89 (0.81-0.98)
Unfavorable	106/35			121/20		
Knowledge						
Not aware	215/56	90.92**	2.62 (2.00-3.43)	241/30	39.94**	1.43 (1.24-1.65)
Aware	39/90			80/49		
Residence						
Rural	171/59	27.48**	1.52* (1.28-1.80)	201/29	17.41**	1.23* (1.11-1.38)
Urban	83/87			120/50		

In terms of willingness to accept DBM, significant positive associations were found with being a graduate (PR=1.33; 95% CI: 1.17-1.50), rural residence (PR=1.23; 95% CI: 1.11-1.38), and lack of awareness (PR=1.43; 95% CI: 1.24-1.65). In contrast, being a housewife (PR=0.84; 95% CI: 0.74-0.95), living in a joint family (PR=0.85; 95% CI: 0.75-0.97), and identifying as Hindu (PR=0.81; 95% CI: 0.70-0.94) were associated with significantly lower likelihood of willingness to accept DBM. Variables not significantly associated with willingness to accept included income, attitude, and number of children.

Mediation analysis: the attitude between knowledge and willingness

**Figure 5:**
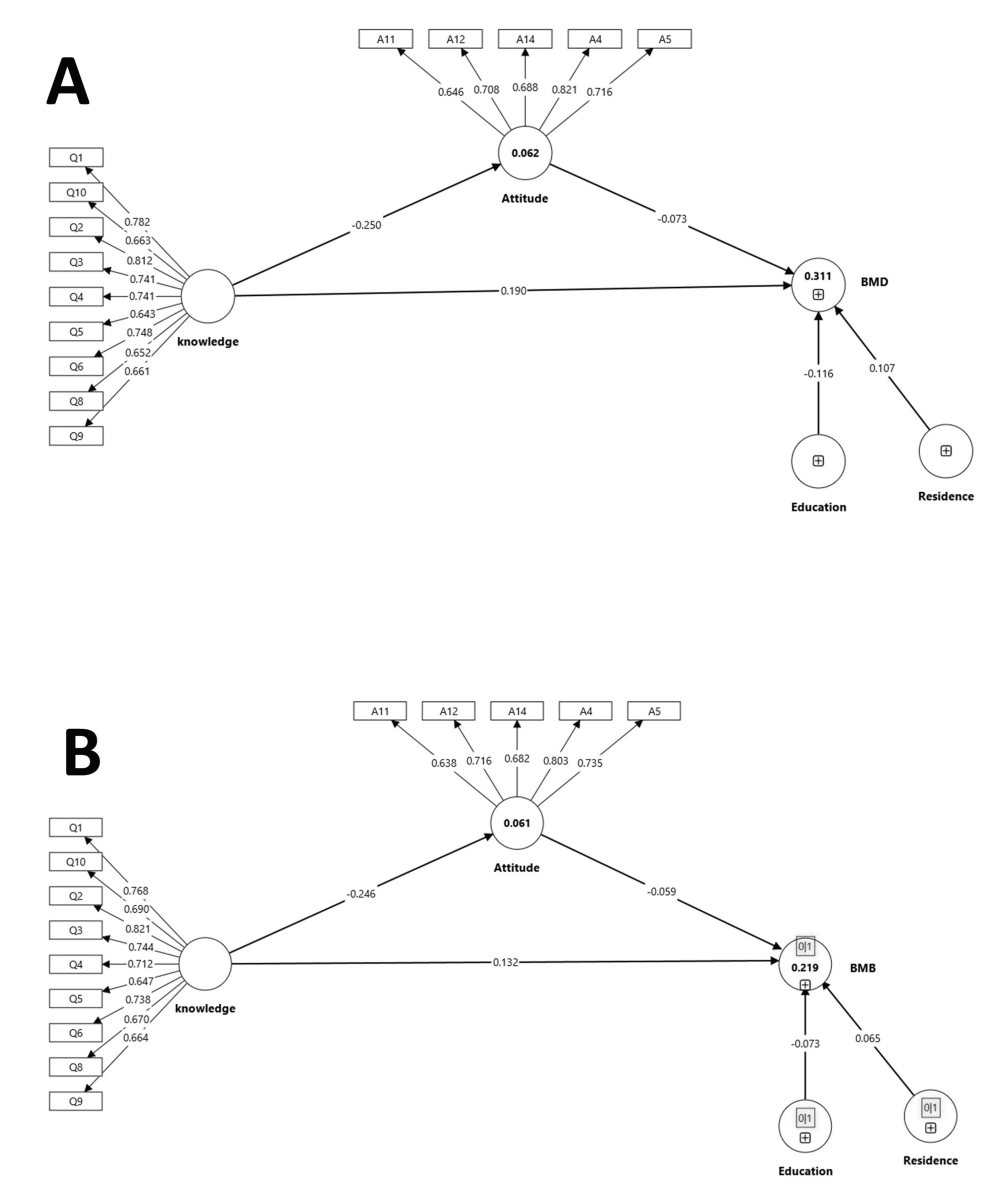
Mediation model showing the direct, indirect, and total effects of knowledge on BMD and DBM acceptance through attitude Panel A illustrates the mediation model for BMD. Panel B illustrates the mediation model for DBM acceptance. The numerical values displayed along the arrows represent standardized path coefficients (β), indicating the strength and direction of the relationships between constructs. The numerical values shown inside the circles represent the coefficient of determination (R²), indicating the proportion of variance explained by the model. BMD: breast milk donation; DBM: donor breast milk Image created by the authors using Microsoft PowerPoint (Microsoft Corporation, Redmond, Washington, United States)

To assess the mediating effect of attitude in the relationship between knowledge and willingness to donate and accept DBM, a mediation analysis was conducted using SmartPLS Version 4.1.1.2. The analysis evaluated the direct, indirect, and total effects among knowledge, attitude, and willingness outcomes, with attitude modeled as the mediator (Table 2, Figure 5).

**Table 2:** Mediation analysis showing the direct, indirect, and total effects of knowledge and attitude on willingness to donate and accept DBM BCa: bias-corrected; CI: confidence interval; B: unstandardized coefficient; BMD: breast milk donation; DBM: donor breast milk

Effect	Predictor	Dependent	B	95% BCa CI	t value	P-value
Direct	Knowledge	BMB	0.132	0.09 to 0.17	6.74	<0.001
	Attitude	BMB	-0.059	-0.09 to -0.02	3.82	<0.001
	Knowledge	Attitude	-0.246	-0.31 to -0.14	5.76	<0.001
Indirect	Knowledge (via attitude)	BMB	0.014	0.007 to 0.024	3.35	0.001
Total	Knowledge	BMB	0.14	0.10 to 0.18	7.38	<0.001
Direct	Knowledge	DBM	0.19	0.15 to 0.23	8.70	<0.001
	Attitude	DBM	-0.073	-0.11 to -0.03	3.63	<0.001
	Knowledge	Attitude	-0.25	-0.34 to - 0.17	5.89	<0.001
Indirect	Knowledge (via attitude)	DBM	0.018	0.008 to 0.02	3.31	<0.001
Total	Knowledge	DBM	0.208	0.16 to 0.24	9.46	<0.001

H1 proposed that mothers with higher knowledge about BMB would be more likely to express willingness for BMD and acceptance of DBM. The findings supported this hypothesis, as knowledge showed a significant positive direct effect on willingness to donate (B=0.19; 95% BCa CI: 0.15-0.23; p<0.001) and willingness to accept DBM (B=0.132; 95% BCa CI: 0.09-0.17; p<0.001).

H2 suggested that favorable attitudes toward BMB would be positively associated with willingness regarding BMD and DBM. However, contrary to expectations, attitude demonstrated a significant negative direct effect on willingness to donate (B=-0.073; 95% BCa CI: -0.11 to -0.03; p<0.001) and willingness to accept DBM (B=-0.059; 95% BCa CI: -0.09 to -0.02; p<0.001), indicating that more favorable attitudes were associated with lower willingness.

H3 proposed that knowledge about BMB would be positively associated with favorable attitudes toward BMB. However, the analysis revealed a significant negative association between knowledge and attitude (B=-0.246; 95% BCa CI: -0.31 to -0.14; p<0.001), suggesting that higher knowledge was associated with less favorable attitudes.

H4 proposed that attitude would mediate the relationship between knowledge and willingness regarding BMD and DBM. The mediation analysis supported this hypothesis, demonstrating significant indirect effects of knowledge on willingness to donate (B=0.018; 95% BCa CI: 0.008-0.02; p<0.001) and willingness to accept DBM (B=0.014; 95% BCa CI: 0.007-0.024; p=0.001), indicating partial mediation.

H5 examined the association of socio-demographic variables with knowledge, attitudes, and willingness regarding BMD and DBM. The findings indicated that education, income, and family type were significantly associated with knowledge, attitudes, and willingness to donate and accept donor milk.

In terms of model evaluation, the reliability and discriminant validity of the constructs were assessed to ensure the robustness of the findings. The outer loadings of the constructs, while not all exceeding the commonly accepted threshold of 0.70, were found to meet acceptable levels for construct reliability, which were above 0.50. This indicates that the items used to measure each construct demonstrated adequate reliability.

Further, the discriminant validity of the model was well established, confirming that the constructs measured in the analysis were sufficiently distinct from one another. This was confirmed through both the HTMT ratio and the Fornell-Larcker criterion. Both methods supported the notion that the constructs in the model did not overlap excessively, indicating that each construct represented a unique aspect of the mothers' knowledge, attitudes, and willingness regarding BMB.

Additionally, the analysis did not reveal any concerns regarding multicollinearity. The highest variance inflation factor (VIF) observed was 2.27, which is well below the threshold of 5. This indicates that the predictor variables in the model were not highly correlated, ensuring that multicollinearity issues did not distort the mediation analysis results.

## Discussion

In line with existing behavioral theories such as the TPB [14] and the Health Belief Model, knowledge was expected to positively influence attitudes and, consequently, behavioral intentions. Our study partly confirmed this expectation: knowledge significantly and positively predicted willingness to donate breast milk (BMD). However, attitude served as a negative predictor, and knowledge negatively predicted attitude, indicating a counterintuitive pathway. Although this finding contrasts with conventional assumptions within the TPB, it may reflect the complex sociocultural and emotional context surrounding BMB in settings where donor milk practices are relatively unfamiliar.

The bivariate correlation confirmed these findings, showing that both knowledge and attitude had a negative relationship with BMD. This may suggest that while individuals may become more knowledgeable about milk donation processes such as donor screening, storage, and safety protocols, this knowledge may simultaneously provoke concerns or discomfort, thereby shaping more hesitant or conflicted attitudes.

One possible explanation for this pattern is "awareness-induced skepticism", where increased knowledge regarding ethical, religious, cultural, or safety-related aspects of human milk banking may heighten caution or ambivalence toward milk donation [16]. Concerns over bodily fluid sharing, disease transmission, or institutional handling of human biological materials may create a disconnect between what one knows and how one feels about donating milk [17]. However, this interpretation should be considered exploratory, as alternative explanations related to measurement characteristics, response patterns, or construct validity cannot be excluded.

Importantly, this relationship persisted even after controlling for education and residence, suggesting the negative impact of knowledge on attitude is not merely due to socio-demographic factors but reflects culturally and emotionally mediated interpretations of milk donation.

Despite the negative path through attitude, knowledge had a significant indirect positive effect on BMD through attitude, indicating partial mediation. This may indicate that although attitudes may become more cautious or conflicted, increased knowledge still supports donation intentions either by overriding emotional resistance or through rational decision-making.

Several studies support these findings. In Mumbai, 67% of mothers had negative attitudes toward milk banking [18], and in Punjab, over half had insufficient knowledge [19]. Meanwhile, in Wuhan, 81.3% of mothers were willing to donate surplus breast milk [20], indicating how cultural context and trust in health systems significantly shape donation behavior. Additionally, international studies from Switzerland and Kenya show that trust in milk bank processes, particularly pasteurization and screening, plays a critical role in willingness to donate [17,21]. Notably, a study based on the TPB also found that while attitude positively predicted donation intention, awareness had a negative influence. This further underscores that the relationship between knowledge, attitude, and willingness is not always linear or uniformly positive, as traditionally assumed in behavioral models [12].

Similarly, knowledge had a significant and positive direct effect on willingness to accept DBM, indicating that informational exposure encourages mothers to consider using donor milk when needed. However, paralleling the donation model, attitude again acted as a negative mediator, and knowledge negatively predicted attitude, suggesting that the more one knows, the more cautious or hesitant they may feel about accepting donor milk.

This again reflects the emotional and cultural complexities surrounding milk banking. Concerns about contamination, trust in handling procedures, or religious implications may outweigh the perceived benefits for some mothers, leading to negative or ambivalent attitudes, even among those who understand the benefits of donor milk [22].

Interestingly, despite the negative influence of attitude, the indirect effect of knowledge on DBM through attitude was significant and positive, indicating that knowledge supports acceptance, even if the emotional response is not uniformly favorable. This is another example of partial mediation, reinforcing the idea that cognitive factors (knowledge) and affective responses (attitude) both shape behavioral intentions, though not always in a linear or expected way.

Comparative research reinforces this dynamic. In New York, 62% of mothers chose formula over donor milk [23], and in Nigeria, 71% were unwilling to accept donor milk due to fears of disease [24]. In contrast, in Switzerland, 85% of potential recipients trusted milk banks, which increased their willingness to accept DBM [21]. Trust in safety and handling procedures consistently appears as a key facilitator.

Our study also aligns with prior findings showing that higher education, income, and employment status are associated with greater acceptance of donor milk. These factors may correspond with better access to reliable information, higher health literacy, and greater trust in healthcare systems. Additionally, mothers with prior experience (i.e., those with children) tend to show higher levels of both knowledge and acceptance, as supported by studies from China and India [19,25].

The findings extend TPB-informed research by suggesting that cognitive and affective components may not operate in a strictly linear manner in culturally sensitive health behaviors. Although TPB proposes that favorable attitudes strengthen behavioral intention, the present study indicates that increased knowledge may activate complex cultural and emotional considerations that shape attitudes in nuanced ways.

Future studies should use robust, multi-item measures and avoid excessive categorization. Including health professionals' perspectives and conducting studies in similar socioeconomic settings may enhance relevance and generalizability. Longitudinal and mixed-method approaches are recommended.

Implications

In neonatal intensive care unit (NICU) settings, counselling should address not only the clinical benefits of DBM but also concerns related to safety, religious beliefs, and family influence. Clear explanations of donor screening and pasteurization processes may help strengthen trust. Involving key family decision-makers, particularly in joint family contexts, may further support acceptance. For BMB services, transparent communication about safety protocols and quality control is essential. Integrating structured antenatal and postnatal education may help normalize discussion around DBM. At the public health level, culturally sensitive awareness initiatives led by healthcare professionals may improve donation and acceptance.

Limitations

This cross-sectional analytical study relied on convenience sampling from a single tertiary care facility, which may limit the generalizability of the findings to broader maternal populations and diverse sociocultural settings. Although mediation analysis was conducted within a TPB-informed framework, the cross-sectional design does not permit the establishment of temporal or causal pathways among knowledge, attitude, and willingness. Therefore, the mediation findings should be interpreted as statistical associations consistent with a mediational framework rather than evidence of causality. The categorization of continuous scores may have reduced variability and obscured nuanced relationships among variables. In addition, willingness to donate or accept DBM was assessed through self-reported hypothetical intentions rather than actual behavior, as no fully operational local BMB was available during the study period. Responses may also have been influenced by social desirability bias, particularly given the sensitive and socially valued nature of breastfeeding and infant care practices. Furthermore, the sample included both antenatal and postnatal mothers, whose differing experiences and exposure to health information may have influenced knowledge, attitudes, and willingness. Although face and content validation of the willingness questionnaire were performed, a more extensive psychometric evaluation of this brief measure may strengthen future research.

## Conclusions

This study identified a paradoxical pattern in which higher knowledge about BMB was associated with less favorable attitudes while also being positively associated with willingness to donate or accept donor milk. Attitude statistically mediated the association between knowledge and willingness, suggesting that knowledge alone may not be sufficient to shape behavioral intentions regarding BMB. These findings highlight the importance of not only improving awareness but also addressing cultural, emotional, and psychological concerns related to donor milk practices. Culturally sensitive educational and counselling interventions may help strengthen trust and improve willingness regarding BMD and acceptance. Further longitudinal and qualitative research is warranted to better understand the complex relationship between knowledge, attitude, and willingness in the context of BMB.

## Appendices

Appendix A

Section A: Demographic Profile

Select the response (√) that best represents your individual profile.

1. Age (in years): a. <18 b. 19-25 c. 26-35 d. >36

2. Residence: a. Urban b. Rural

3. Religion: a. Hindu b. Christian c. Muslim d. Others

4. Marital status: a. Married b. Unmarried c. Divorced d. Widow

5. Education: a. Illiterate b. Primary level education c. Secondary level education d. Higher secondary education e. Graduate f. Postgraduate

6. Occupation: a. Unemployed/housewife b. Government service c. Private service d. Self-employed

7. Family income per month (INR): a. <5,000 b. 5,000-20,000 c. 20,000-50,000 d. >50,000

8. Type of family: a. Joint family b. Nuclear family

9. Number of children: a. 0 b. 1 c. 2 d. ≥3

Section B: Knowledge

Kindly read the question carefully and select the correct answer for each item by putting a tick mark (√).

Q1. Are you aware about breast milk donation? a. Yes b. No

If yes, what is the source of information about breast milk donation? a. Family members/friends/relatives b. Mass media c. Healthcare professionals d. Articles or books

Q2. Are you aware about breast milk bank? a. Yes b. No

If yes, what is the source of information about breast milk bank? a. Family members/friends/relatives b. Mass media c. Healthcare professionals d. Articles or books

Q3. What do you think about breast milk donation? a. Expressing breast milk and feeding one's own baby b. Collection and processing of milk from several nursing mothers c. Refrigerated breast milk d. I don't know

Q4. What do you mean about breast milk bank? a. Service provided for collection, storage, processing, and distribution b. Bank from which mothers can obtain milk c. Both a and b d. None of the above e. I don't know

Q5. Who do you think can donate breast milk to the breast milk bank? a. Mothers having excessive milk b. Healthy lactating mothers c. Mothers not under any medication d. All of the above e. I don't know

Q6. Who do you think cannot donate breast milk to the breast milk bank? a. Mothers on chemotherapy b. HIV-positive mothers c. Mothers who consume alcohol d. All of the above e. I don't know

Q7. How long can expressed breast milk, kept at room temperature, be given to the baby? a. Up to 1 hour b. Up to 8 hours c. Up to 12 hours d. Up to 1 day e. I don't know

Q8. What do you think is the function of a breast milk bank? a. To provide milk to babies admitted in NICU and pediatric wards b. To store milk for a long period of time c. To export and import donated breast milk d. All of these e. None of these f. I don't know

Q9. Whom do you think are the recipients of donor breast milk? a. Premature infants b. Infants with stomach and intestinal anomalies c. Both a and b d. I don't know

Q10. What is the best container to collect breast milk for the breast milk bank? a. Plastic mug b. Wide-mouthed stainless steel c. Polythene bags d. Silicon bags e. I don't know

Q11. Where do you think is the best place to store breast milk for a long time? a. In a room b. Fridge c. Deep freezer d. All of the above e. I don't know

Q12. Do you think the steps mentioned below on the breast milk donation process are in order or not?

i. Donor mother registration and screening ii. Milk expression iii. Pasteurization and testing iv. Storage

a. Yes b. No c. I don't know

Q13. What do you think about the cost of donated breast milk? a. Free b. Paid c. I don't know

Q14. Do you think the consent of the mother is necessary for giving donated milk to her baby? a. Yes b. No

Q15. In what ways are mothers benefited by donating milk? a. Mothers donate free of cost b. Mothers are given incentives c. I don't know

Bottom of the Form

Section C: Attitude of Pregnant and Nursing Mothers on Breast Milk Donation and Breast Milk Bank

Direction:  Kindly check (√) and rate yourself honestly based on what you actually do on the given statements using the following scales:

**Table 3:** Attitude tool

Statement	Strongly agree	Agree	Neutral	Disagree	Strongly disagree
A1. Providing donated breast milk for newborn babies is better than formula feed.					
A2. Donated breast milk can provide the same nutritious value as that of giving one's own mother's milk.					
A3. If a mother is diagnosed with any illness where breastfeeding is contraindicated, donated breast milk is preferred.					
A4. I can donate breast milk if I am having excessive breast milk.					
A5. I would prefer donated breast milk if I am having insufficient breast milk.					
A6. Like a blood bank, having a breast milk bank is essential for each hospital.					
A7. Having a breast milk bank helps the newborn receive donated breast milk at times of non-availability.					
A8. The risk of disease transmission is present in donated breast milk to the baby.					
A9. Donated breast milk may lose its nutritional value compared to a mother's own milk.					
A10. Donated breast milk has negative influences on bonding between child and biological mother.					
A11. Donating breast milk is a culturally and religiously unacceptable practice.					
A12. I feel lazy to feed my own breast milk to my baby if donated breast milk is available.					
A13. Donated breast milk may cause allergic reactions in the baby.					
A14. Feeding donated breast milk to the baby is considered a sin in the community.					

Section D: Willingness

Breast Milk Donation (BMD)

BMD1. If you have excessive milk production during the lactation period, are you willing to donate it to another baby? a. Yes b. No

If no, why? a. Due to sociocultural issues b. Due to transmission of diseases c. Disapproval from family members d. Due to lower self-esteem

Donor Breast Milk Acceptance (DBM)

DBM1. If you have inadequate or ambient breast milk secretion, will you accept the donor breast milk for your baby? a. Yes b. No

If no, why? a. Fear of disease transmission b. Suspecting impurity c. No nutritional value as that of formula feed d. Disapproval of the community e. Due to cultural values

Appendix B

**Table 4:** Assessment of convergent validity and internal consistency reliability for the DBM and BMD models BMD: breast milk donation; DBM: donor breast milk

Variable	Items	Loading	Cronbach's alpha	rho_a	rho_c	AVE
DBM	Attitude					
	A11	0.638	0.773	0.790	0.840	0.514
	A12	0.716				
	A14	0.682				
	A4	0.803				
	A5	0.735				
	Knowledge					
	K1	0.768	0.885	0.907	0.905	0.517
	K2	0.821				
	K3	0.744				
	K4	0.712				
	K5	0.647				
	K6	0.738				
	K8	0.670				
	K9	0.664				
	K10	0.690				
BMD	Attitude					
	A11	0.646	0.773	0.801	0.841	0.516
	A12	0.708				
	A14	0.688				
	A4	0.821				
	A5	0.716				
	Knowledge					
	K1	0.782	0.885	0.921	0.905	0.516
	K2	0.812				
	K3	0.741				
	K4	0.741				
	K5	0.643				
	K6	0.748				
	K8	0.652				
	K9	0.663				
	K10	0.690				

Appendix C

**Table 5:** Discriminant validity at the indicator level (crossloading) for the DBM and BMD models BMD: breast milk donation; DBM: donor breast milk

Variable	Items	Attitude	Knowledge
DBM	A11	0.638	-0.097
	A12	0.716	-0.239
	A14	0.682	-0.154
	A4	0.803	-0.200
	A5	0.735	-0.143
	K1	-0.369	0.768
	K2	-0.248	0.821
	K3	-0.134	0.744
	K4	-0.159	0.712
	K5	-0.113	0.647
	K6	-0.089	0.738
	K8	0.004	0.670
	K9	-0.041	0.664
	K10	-0.219	0.690
BMD	A11	0.646	-0.098
	A12	0.708	-0.246
	A14	0.688	-0.153
	A4	0.821	-0.205
	A5	0.716	-0.138
	K1	-0.373	0.782
	K2	-0.248	0.812
	K3	-0.134	0.741
	K4	-0.159	0.741
	K5	-0.112	0.643
	K6	-0.091	0.748
	K8	0.008	0.652
	K9	-0.041	0.661
	K10	-0.218	0.663

Appendix D

**Table 6:** Discriminant validity at the construct level (heterotrait-monotrait ratio matrix) for the DBM model All the values are less than 0.85; hence, discriminative validity is good. DBM: donor breast milk

Variables	Attitude	DBM	Education	Residence	Knowledge
Attitude	-	-	-	-	-
DBM	0.279	-	-	-	-
Education	0.233	0.269	-	-	-
Residence	0.310	0.209	0.290	-	-
Knowledge	0.254	0.424	0.390	0.171	-

Appendix E

**Table 7:** Discriminant validity at the construct level (Fornell-Larcker criterion) for the DBM model DBM: donor breast milk

Variables	Attitude	DBM	Education	Residence	Knowledge
Attitude	0.717	-	-	-	-
DBM	-0.271	1.000	-	-	-
Education	0.209	-0.269	1.000	-	-
Residence	-0.285	0.209	-0.290	1.000	-
Knowledge	-0.246	0.416	-0.376	0.181	0.719

Appendix F

**Table 8:** Discriminant validity at the construct level (heterotrait-monotrait ratio matrix) for the BMD model All the values are less than 0.85; hence, discriminative validity is good. BMD: breast milk donation; DBM: donor breast milk

Variables	Attitude	DBM	Education	Residence	Knowledge
Attitude	-	-	-	-	-
BMD	0.318	-	-	-	-
Education	0.233	0.332	-	-	-
Residence	0.310	0.262	0.290	-	-
Knowledge	0.254	0.498	0.390	0.171	-

Appendix G

**Table 9:** Discriminant validity at the construct level (Fornell-Larcker criterion) for the BMD model BMD: breast milk donation; DBM: donor breast milk

Variables	Attitude	DBM	Education	Residence	Knowledge
Attitude	0.718	-	-	-	-
DBM	-0.306	1.000	-	-	-
Education	0.205	-0.332	1.000	-	-
Residence	-0.287	0.262	-0.290	1.000	-
Knowledge	-0.250	0.498	-0.379	0.187	0.718
